# Author Correction: Induction of functional tissue-engineered skeletal muscle constructs by defined electrical stimulation

**DOI:** 10.1038/s41598-024-57466-4

**Published:** 2024-03-28

**Authors:** Akira Ito, Yasunori Yamamoto, Masanori Sato, Kazushi Ikeda, Masahiro Yamamoto, Hideaki Fujita, Eiji Nagamori, Yoshinori Kawabe, Masamichi Kamihira

**Affiliations:** 1 Department of Chemical Engineering, Faculty of Engineering, Kyushu University, 744 Motooka, Nishi-ku, Fukuoka, 819-0395 Japan; 2 Graduate School of Systems Life Sciences, Kyushu University, 744 Motooka, Nishi-ku, Fukuoka, 819-0395 Japan; 3 Toyota Central R&D Laboratories Inc., 41-1 Yokomichi, Nagakute, Aichi 480-1192 Japan; 4 Present Address: Laboratory for Comprehensive Bioimaging, Riken Qbic, 6-2-3 Furuedai, Suita, Osaka 565- 0874 Japan; 5 Present Address: Department of Biotechnology, Graduate School of Engineering, Osaka University, 2-1 Yamadaoka, Suita, Osaka 565-0871 Japan

Correction to: *Scientific Reports* 10.1038/srep04781, published online 24 April 2014

This Article contains an error.

In the Methods section

“The total number of nuclei in the tissue construct was counted using NucleoCassette™ and NuceloCounter (Chemometec, Allerød, Denmark).”

should read,

“The total number of nuclei in the tissue construct was counted using NucleoCounter® (ChemoMetec, Allerød, Denmark).”

